# Reviewed article: Bando DH, Neto FC, Queiroz AP. Stroke mortality negatively associated with health, education, and safety: an ecological study, Minas Gerais, 2014-2022. Epidemiol Serv Saude. 2025:34;e20240820

**DOI:** 10.1590/S2237-96222025v34e20240820.a

**Published:** 2025-09-29

**Authors:** Rodrigo Pinheiro de Toledo Vianna

**Affiliations:** 1 Universidade Federal da Paraíba, João Pessoa, PB, Brazil

Peer review A

## Questionnaire

Does the manuscript contain new and significant information to justify publication? No

Does the Abstract (Summary) clearly and accurately describe the content of the article? Yes

Is the problem significant and concisely stated? Yes

Are the methods described comprehensively? Yes

Are the interpretations and conclusions justified by the results? No

Is adequate reference made to other work in the field? Yes

## Manuscript Structure

Length of article is: Adequate

Number of tables is: Adequate

Number of figures is: Adequate

Please state any conflict(s) of interest that you have in relation to the review of this paper. None

Interest: Below Poor

Quality: Average

Originality: Below Average

Overall: Below Average

Recommendation: Major Revision

## Comments

Prezados autores

Parabéns pelo trabalho e pelo detalhado e rigoroso procedimento de análise espacial realizado.

Uma sugestão que me parece oportuna para este trabalho seria encaminhá-lo para publicação em uma revista de estatística espacial, descrevendo a possibilidade e o potencial de uso de informações de saúde em modelagens espaciais.

Com relação aos resultados, apesar de consistentes, eles não me parecem muito conclusivos para serem utilizados como subsídio para tomada de decisão em saúde por serem muito genéricos.

A conclusão de que piores condições de vida estão associadas a maiores taxas de AVC é um achado importante que é largamente confirmada por inúmeros trabalhos, entretanto considero que seria importante contextualizar este achado para o estado de Minas Gerais e assim justificar os resultados encontrados.

Outra coisa que precisaria ser melhor discutida é o fato do indicador de vulnerabilidade não ter associação significativa, uma vez que os autores afirmam, corroborado por muitos estudos que piores condições de vida aumentam as taxas de mortalidade por AVC.

Outro ponto importante que deveria ser discutido, na minha opinião, é a possibilidade de falácia ecológica, ou seja, maiores taxas de Mortalidade em regiões específicas diferenciadas dos municípios. Por haver muitos municípios pequenos, este problema pode ser minimizado, mas é importante considerar esta questão nos estudos ecológicos.

Também, na discussão, me parece que seria importante relacionar os achados dos conglomerados espaciais com os resultados da regressão, especialmente pela dependência espacial observada.

Minha impressão é que os resultados e, especialmente, a discussão está muito descritiva e pouco analítica e isto é o ponto fraco do artigo, pois não traz informações novas ao campo de conhecimento e os achados não dialogam com o contexto do local onde a pesquisa foi realizada.

Pelo rigor metodológico da análise espacial feita, minha recomendação é que este artigo pode ser considerado para a publicação desde que a discussão seja refeita mostrando por que e como a identificação destes conglomerados e destas relações podem subsidiar os gestores na tomada de decisões e na formulação de políticas públicas no campo da saúde, tal como esta colocado no final da sua conclusão.

